# Temporal Artery Biopsy: A 10-Year Multi-centre Review of Current Practice

**DOI:** 10.7759/cureus.98670

**Published:** 2025-12-07

**Authors:** Adrian J Tam, Samantha Leng, Christopher Steen, Jonathan Chua

**Affiliations:** 1 General Surgery, Eastern Health, Ringwood, AUS; 2 General Surgery, Cabrini Health, Melbourne, AUS

**Keywords:** general and vascular surgery, giant cell arteritis (gca), targeted biopsy, temporal artery biopsy, vasculitis diagnosis

## Abstract

Background and objective

Surgical teams are commonly requested to perform temporal artery biopsies (TAB) to assist in the diagnosis of giant cell arteritis (GCA). This study aimed to assess the clinical utility of TAB and its effect on the subsequent medical management of GCA.

Methods

We conducted a 10-year retrospective analysis of all patients who underwent TAB between 2011 and 2021 at three hospitals of a health network in Melbourne. Patient characteristics included accepted risk factors per the American College of Rheumatology (ACR), and whether treatment had been initiated before surgery. Surgical variables included biopsy specimen length, histopathology findings, and the mean time to theatre.

Results

Data were collected for 216 eligible patients, of whom 174 (80.6%) had commenced steroids before TAB. Forty patients (18.5%) had positive TAB results on histopathology. A significant proportion of patients (37.3%, n = 53) with negative TAB results completed their course of steroid therapy for GCA (p<0.05) despite negative histopathology. In addition, 32 patients (14.9% of total patients) underwent TAB despite pre-op documentation of the intention to continue steroid therapy regardless of biopsy results. There were no statistically significant factors identified between patient groups who continued or ceased steroid treatment post TAB, including TAB sample length. Colour Doppler ultrasound was only used in 6.98% of our study population.

Conclusions

The utility of TAB in guiding medical management of GCA appears to be limited. A significant proportion of patients completed a full course of steroid therapy, irrespective of histopathology. Furthermore, 14.9% of patients underwent TAB despite pre-documented plans to continue steroid use regardless of the result. Further research is needed to update current clinical probability scores to include rigorous parameters to guide clinicians regarding the cessation of steroids.

## Introduction

Giant cell arteritis (GCA) is the most common form of systemic vasculitis in adults over the age of 50, with incidence peaking between the ages of 70 and 80 [1]. Complications from untreated GCA, most notably partial or complete loss of vision, can be catastrophic and permanent. Thus, early recognition of symptoms and timely initiation of treatment are paramount. The American College of Rheumatology (ACR) 1990 guidelines established a set of clinical criteria for classifying GCA, with the presence of three or more criteria considered confirmatory [2]. The ACR criteria were recently updated with the addition of several new clinical criteria and the introduction of a weighted scoring system to address its relatively low sensitivity [2,3,4]. In collaboration with the European Alliance of Associations for Rheumatology (EULAR), the 2022 ACR/EULAR criteria were published, introducing age >50 years as a mandatory criterion, integrating imaging findings, and assigning new weights to each feature. A total score >six now allows classification of GCA [3]. 

Although temporal artery biopsy (TAB) carries the greatest weighting in the ACR/EULAR criteria, it is still widely accepted that a negative TAB result does not exclude GCA due to the possibility of false negatives [5,6,7,8]. Neither the ACR nor the EULAR criteria consider positive TAB a mandatory criterion. Despite this, TAB is still routinely performed in cases where GCA is suspected. Several studies have shown that most patients undergoing TAB already meet the confirmatory ACR criteria at the time of biopsy [9,10]. In addition, the ACR recommends that patients with high suspicion of GCA start early first-line steroid treatment to avoid permanent disability, limiting the role of TAB in initial management decisions for these patients.

Since the publication of the 1990 ACR guidelines, colour Doppler ultrasound (CDUS) has been validated for detecting a non-compressible vessel, known as the “Halo sign,” and has been shown to have sensitivity comparable to TAB in diagnosing GCA [4,11,12,13]. This offers a less invasive and less resource-intensive alternative to TAB. The development of highly sensitive clinical diagnostic criteria, along with the development of non-invasive radiological adjuncts, raises questions regarding the true utility of TAB in the diagnosis and subsequent management of GCA. Thus, this study aimed to assess the current clinical utility of TAB and its effect on the medical management of patients with suspected GCA. Although the gold standard for diagnosis remains physician opinion at six months, the criteria outlined by ACR/EULAR remain critical in guiding initial clinical suspicion and are therefore relevant for the purposes of this study.

Despite TAB being a procedure with low morbidity, potentially fatal or disabling complications such as ischaemic stroke, scalp or tongue necrosis, and frontalis dysfunction due to facial nerve injury have been reported, in addition to less morbid but relatively more common complications such as bleeding, haematoma, infection, and wound dehiscence [14]. Therefore, the potential benefits of TAB must be weighed against these risks. With this in mind, our primary objective was to evaluate the clinical utility of TAB by examining its impact on steroid management.

## Materials and methods

Study design

A 10-year retrospective chart review was conducted involving all patients who underwent TAB in three acute hospitals of a single health network in Melbourne between 2011 and 2021. Of the 237 patients initially identified, 22 were excluded due to incomplete clinical data, loss to follow-up, or unavailable histology. Patients were identified by the pathology department, and clinical data were then obtained through the health network's electronic medical records.

Data collected included patient age, sex, biopsy side(s), biopsy length, time to biopsy, pre- and postoperative steroid use, operative complications, and CDUS results (when available). Biopsy side(s) and length of biopsy (in millimetres) were obtained from pathology reports. Time to biopsy was defined as the number of days between referral for TAB and the procedure. Patient symptoms and laboratory findings, as specified in the ACR clinical diagnostic criteria [3], were also recorded (Table 1). For patients without a documented ACR score in their notes, a score was calculated using the available clinical information.

**Table 1:** ACR/EULAR classification criteria for GCA* ^*^ACR/EULAR classification criteria are free for use. ^**^Alternative diagnosis should be excluded before applying the criteria [3] Ponte C, Grayson PC, Robson JC, et al.: 2022 American College of Rheumatology/EULAR classification criteria for giant cell arteritis. Arthritis Rheumatol. 2022, 74:1881-9. DOI: 10.1002/art.42325 ACR: American College of Rheumatology; EULAR: European Alliance of Associations for Rheumatology; GCA: giant cell arteritis; TAB: temporal artery biopsy; CDUS: colour Doppler ultrasound; ESR: erythrocyte sedimentation rate; CRP: C-reactive protein; FDG-PET: fluorodeoxyglucose-positron emission tomography

ACR/EULAR criteria	
Mandatory criteria: age >50 years	
Clinical criteria	
Shoulder or neck morning stiffness	2
Sudden visual loss	3
Jaw and or tongue claudication	2
New temporal headache	2
Scalp tenderness	2
Diminished/absent pulse, tenderness, or cord-like appearance of the temporal artery	2
Laboratory/imaging/histological criteria	
Positive TAB or Halo sign on CDUS	5
Maximum ESR >50 mm/hr or CRP >10 mg/L^**^	3
Bilateral axillary involvement on angiography, ultrasound, and PET	2
FDG-PET activity throughout the aorta	2

Primary outcomes included whether steroid treatment was altered by biopsy result, where commencement was defined as at least 60-80 mg of prednisolone daily, and treatment cessation was defined as weaning or cessation of steroids before completing the standard four-week course. Although standard treatment length can extend into years, the four-week threshold was used because the British Society for Rheumatology identifies this as the minimum duration of initial therapy before tapering should begin [15]. Secondary outcomes evaluated the correlation between ACR criteria and TAB results.

Statistical analysis

Comparisons between negative and positive biopsy groups concerning demographics, use of anaesthetic, complications, biopsy lengths, time to biopsy, complications, and steroid use were performed using Fisher's exact test, t-test, and Mann-Whitney U test. The association between ACR criteria, biopsy length, and TAB result was analyzed with Fisher’s exact test, as well as univariate and multivariate logistic regression. As the study inclusion period predated the amendments to the ACR/EULAR criteria, the additional clinical parameters introduced were not captured in clinical documents and hence could not be included in our analysis. However, data were reanalyzed with age removed as an additive criterion in accordance with the updated ACR/EULAR criteria. Given all the criteria in the 1990 ACR are present in the new 2022 ACR/EULAR score, an ACR score >3 is equivalent to an ACR/EULAR score of >6. Thus, our endpoint of 1990 ACR >3 remains a valid endpoint. Statistical analysis was conducted using Stata 18 (StataCorp, College Station, TX).

## Results

A total of 215 patients underwent TAB at our institution between 2011 and 2021 (Table 2). Both TAB-positive and TAB-negative groups had comparable median age (73.44 vs. 73.95 years, respectively, p = 1.0) and gender distribution (70.3% female to 29.7% male in the negative biopsy group, and 75% female to 25% male in the positive group, p = 0.699). The two groups did not differ statistically with respect to the characteristics measured in Table 2. Several patients underwent bilateral biopsies, while others underwent unilateral biopsies. Comparison of biopsy length was conducted in a per-biopsy manner rather than per patient manner. Among the 215 biopsies conducted, 40 (18.6%) were positive for GCA. Notably, 52.5% of patients with positive TAB and 44.6% of those with negative TAB had an ACR score of >3. Of the patients with positive TAB, 21 had an ACR score of >3, and nine of these patients were treatment-naïve until biopsy results were reviewed. Only a small proportion of patients underwent preoperative CDUS (6.98%, 15 of 215 patients).

**Table 2:** Surgical and treatment endpoints ^*^Some biopsies were bilateral: negative biopsy n = 268, while positive biopsy n = 65 CDUS: colour Doppler ultrasound

Variable		Negative biopsy (n = 175)	Positive biopsy (n = 40)		P-value	Test statistic	Test
Age, years, mean		73.44	73.95		0.717	T = 0.362	T-test
Gender, n (%)							
Female		123 (70.3%)	30 (75.0%)		0.699	-	Fisher’s exact
Male		52 (29.7%)	10 (25.0%)				
Anaesthetic modality (n = 189)							
General anaesthetic		58 (33%)	16 (40%)		0.418	-	Fisher’s exact
Sedation		73 (41.7%)	13 (32.5%)				
Local anaesthetic only		22 (12.6%)	7 (17.5%)				
Biopsy length, mm		15.24	16.94		0.121^*^	T = 1.55	T-test
Time to Bx, days		6	5		0.984	U = 2375	Mann-Whitney U
Complications		4	1		0.999	-	Fisher’s exact
Steroid started pre-biopsy							
Yes		142 (81.1%)	31 (77.5%)		0.659	-	Fisher’s exact
No		33 (18.9%)	9 (22.5%)		0.659	-	Fisher’s exact
Pre-op steroid, days							
Mean		6.3	4.9		0.548	T = 0.601	T-test
Steroid continued post Bx		53 (37.3%)	40 (100%)		-		
Steroid ceased post Bx		89 (62.7%)	0		-		
CDUS, n (%)	13 (7.4%)			2 (0.05%)	-		
CDUS positive, n (%)	3 (23.1%)			1 (50.0%)	-		

Pre-biopsy steroid initiation was not significantly different between TAB-positive and TAB-negative groups, with rates of 81.1% vs. 77.5%, respectively (p = 0.4594). In the overall patient cohort, pre-TAB steroids were started in 173 (80.1%), of which 169 had an ACR score of 3 or more. Among the 142 TAB-negative patients in whom pre-biopsy steroid use was recorded, 53 (37.3%) completed their course of steroid therapy for GCA, while 62.7% had their therapy ceased.

Additionally, we found 28 out of the 142 TAB-negative patients who received pre-biopsy steroid management had documented plans to continue steroid therapy regardless of biopsy result. In this “no intention to stop” steroids group, 26 (92.9%) indeed continued steroid therapy for a minimum of four weeks before weaning, while two patients had their steroids weaned or ceased immediately once their biopsies were confirmed to be negative. In contrast, 87 (78%) of the remaining 114 patients had their biopsies stopped or weaned immediately after a negative biopsy. In the group where there was no intention to stop steroid management, having an ACR >3 was 3.4 times higher (p = 0.013). While there were fewer cases of visual symptoms in the "no intention to stop" group (p = 0.0005), other clinical findings, including jaw claudication, new headache, ESR, and temporal artery examination, were not statistically different (Table 3).

**Table 3:** ACR criteria in TAB-negative patients receiving pre-biopsy steroids ACR: American College of Rheumatology; TAB: temporal artery biopsy; ESR: erythrocyte sedimentation rate

Variable	No intention to stop	Intention to stop	Odds ratio	P-value	Statistical test
ACR >3					
No; yes	10 (35.7%); 18 (64.3%)	16 (14.2%); 98 (85.8%)	3.4	0.013	Fisher’s exact
Visual changes					
No; yes	25 (89.3%); 3 (10.7%)	62 (54.9%); 52 (45.1%)	6.99	0.0005	Fisher’s exact
Jaw claudication					
No; yes	27 (96.4%); 1 (3.6%)	96 (84.1%); 18 (15.9%)	-	0.122	Fisher’s exact
ESR >50					
No; yes	12 (42.9%); 16 (57.1%)	40 (64.6%); 74 (35.4%)	-	0.513	Fisher’s exact
Abn examination					
No; yes	17 (60.7%); 11 (39.3%)	71 (61.9%); 43 (38.1%)	-	0.999	Fisher’s exact
New headache					
No; yes	2 (7.1%); 26 (92.9%)	16 (14.2%); 98 (85.8%)	-	0.527	Fisher’s exact

Sedation was the most common anaesthetic modality (n = 86, 40%), while general anaesthesia was utilized in 74 patients (33%), and local anaesthesia was only used in 29 patients (13%). Mean biopsy length and rates of TAB positivity did not appear to be influenced by the type of anaesthesia utilized. Anaesthetic modality was not clearly recorded in 22 biopsy-negative patients and four biopsy-positive patients. Three patients in the biopsy-negative group did not have pre-biopsy steroid status recorded, while post-biopsy steroid use was only recorded in 142 of the 173 patients in whom steroids were started preoperatively.

There were six complications documented, including one case each of nerve injury, wound infection, and postoperative delirium, as well as three cases of non-arterial biopsy (Table 4).

**Table 4:** Postoperative complications

Complication	Number
Nerve injury	1
Wound infection	1
Delirium	1
Non-arterial biopsy	3
Vein	1
Parotid	1
Other tissue	1

Of the variables within the ACR diagnostic criteria (Table 5), only the presence of jaw claudication was associated with a positive TAB result (p < 0.001). The presence of any other clinical variables in isolation did not appear to be associated with a positive TAB result.

**Table 5:** ACR criteria* in TAB-positive and TAB-negative patients ^*^[3] ACR: American College of Rheumatology; TAB: temporal artery biopsy; ESR: erythrocyte sedimentation rate

Variable	Negative	Positive	P-value	Statistical test
ACR >3, n (%)				
No	97 (55.4%)	19 (47.5%)	0.384	Fisher’s exact
Yes	78 (44.6%)	21 (52.5%)		
Visual changes, n (%)				
No	106 (63.9%)	23 (60.5%)	0.712	Fisher’s exact
Yes	60 (36.1%)	15 (39.5%)		
Jaw claudication, n (%)				
No	141 (85.5%)	15 (39.5%)	<0.001	Fisher’s exact
Yes	24 (14.6%)	23 (60.5%)		
ESR >50, n (%)				
No	65 (38.7%)	15 (39.5%)	>0.999	Fisher’s exact
Yes	103 (61.3%)	23 (60.5%)		
Examination, n (%)				
Normal	107 (64.5%)	21 (55.3%)	0.353	Fisher’s exact
Abnormal	59 (35.5%)	17 (44.7%)		
New headache, n (%)				
No	25 (15.1%)	9 (23.7%)	0.228	Fisher’s exact

Furthermore, on univariable and multivariable analysis, jaw claudication was associated with a markedly increased likelihood of GCA, with odds ratios of 9.01 and 8.55, respectively (Table 6).

**Table 6:** Univariable and multivariable logistic regression for factors affecting results For multivariate model: classification accuracy = 83.2%; area under ROC curve = 0.76; Hosmer–Lemeshow test (Chi2 (8) = 11.95, P-value = 0.154) confirmed the model adequacy OR: odds ratio; CI: confidence interval; American College of Rheumatology; ESR: erythrocyte sedimentation rate; ROC: receiver operating characteristic

Variables	Univariate		Multivariate	
	OR (95% CI)	P-value	OR (95% CI)	P-value
Sex (male vs. female)	1.23 (0.56-2.72)	0.608	1.08 (0.44-2.70)	0.861
ACR >3	3.12 (0.91-10.74)	0.072	2.12 (0.44-10.20)	0.348
Visual symptoms	1.15 (0.56-2.38)	0.701	1.16 (0.48-2.81)	0.750
Jaw claudication	9.01 (4.12-19.68)	<0.001	8.55 (3.60-20.36)	<0.001
ESR >50	0.97 (0.47-1.99)	0.929	1.00 (0.41-2.44)	0.995
Abnormal exam	1.47 (0.72-3.00)	0.292	1.19 (0.51-2.78)	0.680
New headache	0.57 (0.24-1.35)	0.202	0.43 (0.14-1.31)	0.139
Avg Bx length, mm	1.02 (0.98-1.07)	0.346	1.02 (0.96-1.07)	0.550

## Discussion

The diagnostic challenges, ramifications from misdiagnosis, and subsequent delay in treatment underscore the rationale for commencing empirical steroid treatment for GCA before TAB. Our study revealed that over a third of patients (37.3%) with a negative TAB completed a course of steroid therapy, while nearly half of patients (46%) already fulfilled the ACR criteria for initiation of empirical management before TAB. The dilemma that arises from this clinical predicament formed the basis for our study. 

Despite the reported high sensitivity and specificity of the initial ACR criteria, subsequent studies have suggested a much lower sensitivity of 37.1% [3]. The limitations imposed by an unreliable set of clinical criteria may have resulted in a sustained demand for TAB in the evaluation of patients with suspected GCA. Recognition of the limitations of the initial ACR guidelines led to the recent amendment in 2022; however, clinical symptoms of GCA remain varied between individual patients. Jaw claudication has been reported in several publications as particularly consistent with positive confirmed GCA [10,16]. In alignment with these findings, our study demonstrated an odds ratio of 9.01 for a positive TAB when jaw claudication was documented. We therefore agree with prior research that clinical suspicion for GCA should remain high when patients present with jaw claudication.

Our study found that 32 patients (14.9% of total patients) underwent TAB despite preoperative documentation indicating the intent to continue steroid therapy irrespective of biopsy results. When comparing clinical criteria between the “no intention to stop steroids” group and the remaining patients who received pre-biopsy steroids (Table 3), the former demonstrated higher odds of having an ACR score >3 (OR: 3.4, p = 0.013). Nevertheless, we were unable to determine which specific clinical features led clinicians to maintain such a high level of suspicion for GCA that they planned to continue steroid treatment irrespective of biopsy findings.

Analysis of individual ACR criteria showed that only visual disturbance differed significantly between groups; however, this finding was paradoxical, as visual disturbance was nearly seven times less common in the “no intention to stop” group (OR: 6.99, p = 0.0005). One rationale to continue steroids regardless of biopsy results may be related to concerns about relapsing or refractory disease, as a positive TAB may be used to support the use of other treatment modalities, such as biologics, in steroid-refractory GCA. The commencement of biologic agents currently mandates histological support for GCA; however, in our retrospective review, the potential use of biologics was mentioned in only two patients, and no patient moved beyond steroid therapy. As the use of biologics becomes more standardized in GCA treatment, confirmatory procedures may play a larger role. 

Another factor contributing to the continuation of steroid therapy despite biopsy results is the level of clinical suspicion. GCA remains a clinical diagnosis, and studies have shown that physicians are skilled at accurately suspecting the disease without the need for biopsy [5,10]. Thus, it is reasonable to maintain steroid therapy in patients where clinical suspicion is high, regardless of biopsy result. This highlights the uncertain benefit of TAB in these patients in whom pre-biopsy clinical suspicion of GCA is already high. 

The sensitivity of TAB has improved over the years. Previous studies have reported false-negative rates as high as 44% [17,18], while advances in pathology and surgical technique have reduced reported false-negative rates to as low as 7% [5,6,7,8]. However, when balanced against the risk of possible permanent disability from missed diagnosis, even this improved false-negative rate is considered by many to be unacceptably high to rely on TAB as a rule-out test. The current ACR guidelines conditionally recommend further investigation of TAB-negative patients with follow-up imaging in the form of ultrasound, MRI, PET, or CT [19]. In cases where biopsy and imaging remain negative, but clinical suspicion remains high, EULAR recommends ongoing management with steroids [4].

Of the 40 TAB-positive patients, nine had steroid management commenced only after their TAB confirmed the GCA, which highlights the importance of the procedure. However, this also means 77.5% of GCA patients potentially underwent a procedure that had no impact on their medical management with steroids. A positive biopsy alone is insufficient to meet ACR/EULAR classification criteria for GCA and should not serve as the sole justification for initiating treatment; likewise, a negative biopsy should not by itself lead to changes in management.

Our study reported a low complication rate of 2.8%, including one case of facial nerve injury. There exists a recognized anatomical danger zone whose vertices are a point 2 cm above the superior orbital rim (point A), the tragus of the ear (point B), a point that is horizontal to point A and superior to point B (point C), and the junction between the zygomatic arch and lateral orbital rim (point B) [20]. A survey of ophthalmology trainees regularly performing TAB independently across multiple hospitals in England found that although 61% of trainees were aware that the anatomical danger zone existed, wherein the facial nerve is more likely to be damaged, 97% still chose their incisions within this zone [21]. Although the TAB procedure is generally considered safe and associated with relatively low morbidity, procedure-specific and anaesthetic-associated risks remain, and hence should be performed judiciously in cases where a positive or negative result may alter the course of treatment for selected patients.

Finally, we attempted to evaluate the use of CDUS in our study as well. However, CDUS was only requested in 15 of our 215 (6.98%) patients. The sparse utilization of CDUS in our study sample precludes a subgroup analysis to support or refute the findings from prior studies evaluating the sensitivity of CDUS. However, this extremely low use of CDUS itself is significant and suggests a gap between current guidelines and real-world practice. The reliability of CDUS is highly operator-dependent and varies with the experience and expertise of the centre performing the examination [11,22]. This latter limitation of CDUS will likely be the main restrictive barrier preventing centres from transitioning from TAB to CDUS going forward. 

Limitations

Our study has certain limitations. Firstly, as a retrospective chart review, patient characteristics may have been missed due to incomplete documentation. In particular, data on post-biopsy steroid use were missing in 17.9% of the 173 patients in whom pre-biopsy steroid use was recorded. This may have introduced bias into our results. Additionally, no patients had the ACR score explicitly documented in patient records. Instead, the ACR scores were retrospectively calculated from documented history and examination findings at the time of admission as well as the time of referral to surgeons for TAB. Additionally, it remains difficult to determine from our data why some patients were designated to continue steroid therapy regardless of biopsy outcome. A prospective study would likely be necessary to accurately explore this decision-making process.

Secondly, our methodology limited inclusion to patients who underwent TAB, rather than all patients admitted with suspected GCA. This approach was selected because surgical codes are reliably recorded in the medical records of patients undergoing procedures during admission, enabling precise identification of this specific patient cohort. Conversely, the utility of a clinical diagnosis of GCA as the main study inclusion criterion may have yielded a more heterogeneous cohort due to heavy reliance on hospital medical officers documenting a clinical diagnosis of GCA on patients’ final discharge summaries, which may occasionally be overlooked in comorbid patients, requiring simultaneous management of multiple concurrent issues. Therefore, it is highly likely that this study did not include a cohort where GCA was suspected and treated without the utilization of a TAB.

## Conclusions

TAB remains a valuable test, as a positive biopsy can bolster clinical suspicion of GCA, while a negative biopsy can cast doubt on patients where clinical suspicion is low or equivocal. It is important to note that TAB by itself is not diagnostic of GCA. Hence, while the procedure is generally safe and carries a low risk of complications, it may be preferable to use TAB more selectively, limiting it to cases where genuine diagnostic uncertainty exists. In particular, TAB is likely most beneficial for patients with a high clinical suspicion of GCA despite low EULAR/ACR scores, and may be unnecessary for those who have both a high clinical suspicion and a high EULAR/ACR score. The use of CDUS offers a tempting alternative to TAB. However, the need for centre experience and expertise in the provision and interpretation of CDUS will potentially limit its effectiveness in the near future, especially in non-specialist centres. Further research is needed to evaluate whether the new clinical probability scores and possible uptake of CDUS will affect the utilization of TAB.
